# Cloning of ε-poly-L-lysine (ε-PL) synthetase gene from a newly isolated ε-PL-producing *Streptomyces albulus* NK660 and its heterologous expression in *Streptomyces lividans*

**DOI:** 10.1111/1751-7915.12108

**Published:** 2014-01-14

**Authors:** Weitao Geng, Chao Yang, Yanyan Gu, Ruihua Liu, Wenbin Guo, Xiaomeng Wang, Cunjiang Song, Shufang Wang

**Affiliations:** 1 State Key Laboratory of Medicinal Chemical Biology, Nankai UniversityTianjin, 300071, China; 2 Key Laboratory of Molecular Microbiology and Technology for Ministry of Education, College of Life Sciences, Nankai UniversityTianjin, 300071, China

## Abstract

ε-Poly-L-lysine (ε-PL), showing a wide range of antimicrobial activity, is now industrially produced as a food additive by a fermentation process. A new strain capable of producing ε-PL was isolated from a soil sample collected from Gutian, Fujian Province, China. Based on its morphological and biochemical features and phylogenetic similarity with 16S rRNA gene, the strain was identified as *Streptomyces albulus* and named NK660. The yield of ε-PL in 30 l fed-batch fermentation with pH control was 4.2 g l^−1^ when using glycerol as the carbon source. The structure of ε-PL was determined by nuclear magnetic resonance (NMR) and matrix-assisted laser desorption/ionization–time of flight mass spectrometry (MALDI-TOF MS). Previous studies have shown that the antimicrobial activity of ε-PL is dependent on its molecular size. In this study, the polymerization degree of the ε-PL produced by strain NK660 ranged from 19 to 33 L-lysine monomers, with the main component consisting of 24–30 L-lysine monomers, which implied that the ε-PL might have higher antimicrobial activity. Furthermore, the ε-PL synthetase gene (*pls*) was cloned from strain NK660 by genome walking. The *pls* gene with its native promoter was heterologously expressed in *Streptomyces lividans* ZX7, and the recombinant strain was capable of synthesizing ε-PL. Here, we demonstrated for the first time heterologous expression of the *pls* gene in *S. lividans*. The heterologous expression of *pls* gene in *S. lividans* will open new avenues for elucidating the molecular mechanisms of ε-PL synthesis.

## Introduction

Two amino acid homopolymers are found in nature (Oppermann Sanio and Steinbuchel, 2002): γ-polyglutamic acid (γ-PGA) and ε-poly-L-lysine (ε-PL). ε-PL consists of 25–35 L-lysine residues with linkages between α-carboxyl groups and ε-amino groups. Because of its safety, biodegradability and antimicrobial activity, it is widely used as a natural food preservative in Japan, Korea, the United States and other countries. The antimicrobial activity of ε-PL is dependent on its molecular size (Shima *et al*., 1984). ε-PL with more than nine L-lysine residues severely inhibits microbial growth. The polymerization mechanisms involved in the chain-length diversity of ε-PL are of particular interest.

Microbial production of ε-PL has been well documented. ε-PL was found in the culture filtrate of *Streptomyces albulus* 346 (preserved as *S. albulus* NBRC 14147), isolated from soil (Shima and Sakai, 1977; 1981). *S. albulus* produces and secretes ε-PL efficiently in the culture medium at pH 4.2–4.5, while ε-PL is immediately degraded by this strain at pH 5.0–8.0 (Kahar *et al*., 2001). In Japan, ε-PL has already entered the commercial market and is produced industrially by fermentation using a mutant derived from *S. albulus* (Hiraki, 2000). A sensitive and simple method for isolating ε-PL-producing strains was developed based on the interaction between the dye and the cationic polymers (Nishikawa and Ogawa, 2002). In recent years, several ε-PL-producing strains were isolated, such as *Streptomyces griseofuscus* (Li *et al*., 2011), *Streptomyces* sp. M-Z18 (Chen *et al*., 2011), *Streptomyces* sp. GIM8 (Liu *et al*., 2011) and *Kitasatospora* sp. PL6-3 (Ouyang *et al*., 2006).

Recently, the molecular mechanisms of microbial synthesis of ε-PL have also been researched. Yamanaka and colleagues (2008) cloned the ε-PL synthetase gene (*pls*) responsible for the synthesis of ε-PL from an ε-PL-producing strain of *S. albulus*, and an ε-PL synthetase (Pls; 130 kDa) was purified from the strain. Pls was found to be a membrane protein with adenylation and thiolation domains characteristic of the nonribosomal peptide synthases (NRPSs). Pls catalyzed L-lysine polymerization using free L-lysine polymer (or monomer in the initial reaction) as acceptor and Pls-bound L-lysine as donor, directly yielding chains of diverse length. Thus, Pls is a new single-module NRPS having an amino acid ligase-like catalytic activity for peptide bond formation.

*Streptomyces lividans* has several features that make it an ideal host for the expression of heterologous recombinant proteins, such as the well-established genetic manipulation procedures, the absence of extensive restriction modification systems, very low endogenous extracellular proteolytic activity, the avoidance of the formation of inclusion body and the suitability of the expression of those genes with a high GC content (Binnie *et al*., 1997; Ziermann and Betlach, 1999; Nakashima *et al*., 2005). Genes of actinomycetes often have high GC content and the preferred codons are not correlated with the abundance of cognate transfer RNAs available in *Escherichia coli* (Gustafsson *et al*., 2004), leading to low-level expression of target genes in *E. coli*. An alternative strategy is to express genes of actinomycetes in *S. lividans*. The heterologous expression of the *pls* gene in *S. lividans* is helpful not only for the improvement of ε-PL yield but also for elucidating the biosynthetic mechanisms of ε-PL in cells. So far, there are no reports of the heterologous expression of the *pls* gene in *S. lividans*.

In this study, a new ε-PL-producing strain was isolated from soil and identified as *S. albulus*. Production of ε-PL by this strain was conducted in 30 l fed-batch fermentation with pH control. Furthermore, the ε-PL synthetase gene was cloned from this strain and heterologously expressed in *S. lividans*.

## Results and discussion

### Isolation and identification of strain NK660

**Figure 1:**
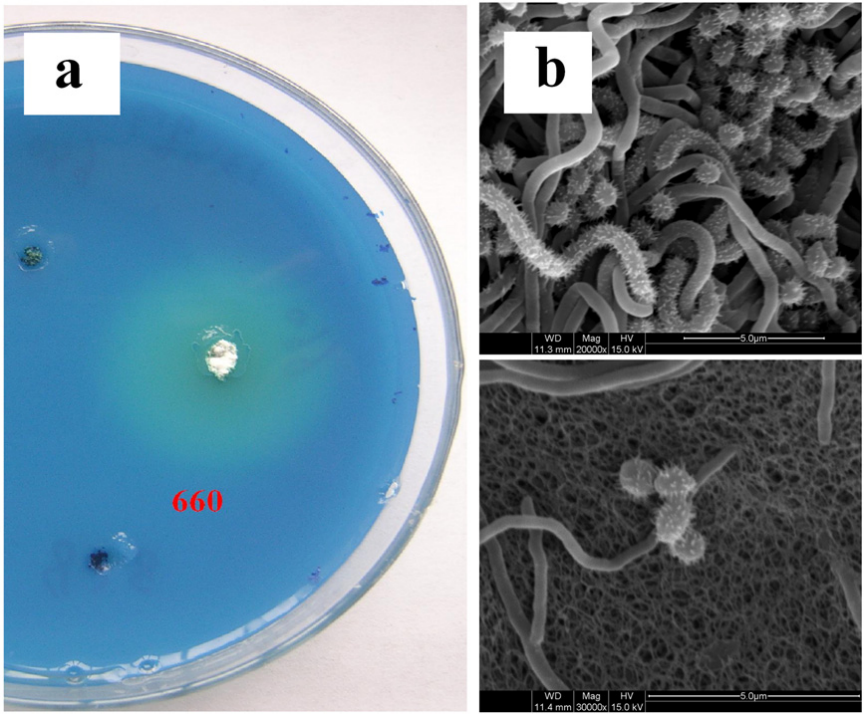
A. The transparent circles formed around colonies of *S. albulus* NK660 in a two-layer agar.B. The electron microscopy photograph of the mycelium and spores of *S. albulus* NK660.

The abundance of actinomycete strains in the soil samples from regions with relatively rich vegetation was significantly higher than usual background levels. A number of actinomycete strains were isolated from the soil samples of Gutian, Fujian, China. One strain (named NK660) showed a clear transparent ring on the methylene blue screening plates after 72 h of incubation and reacted positively with Dragendorff reagent (Fig. 1A), which suggested that strain NK660 might produce ε-PL.

**Figure 2:**
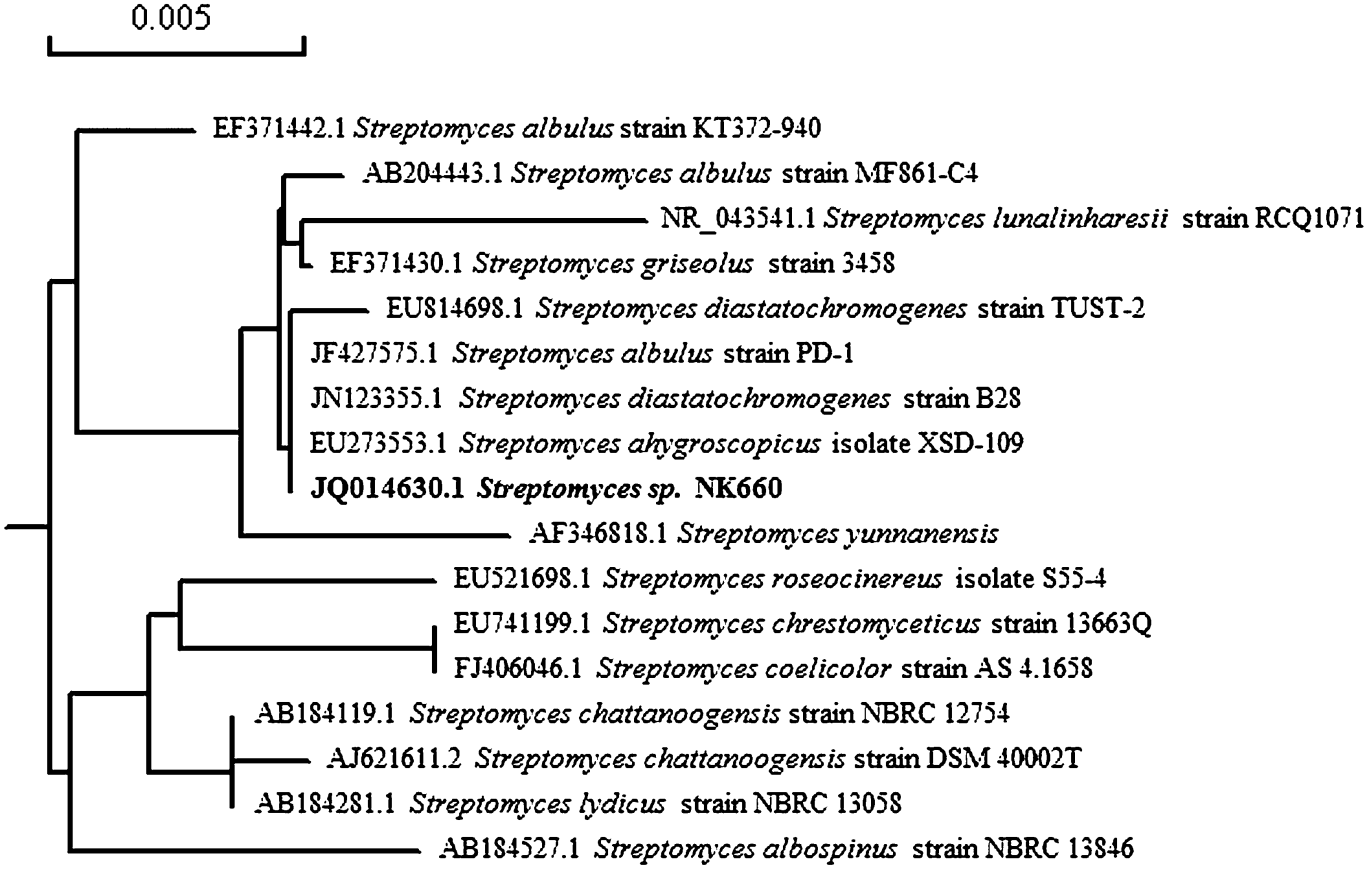
Phylogenetic relationship of *Streptomyces* sp. NK660 (bold fonts) and other *Streptomyces* strains based on the neighbour-joining tree analysis of 16S rRNA gene. The bar (0.005) at the top of the tree indicates the substitution per nucleotide position. GenBank accession numbers are given in front of the strains.

The 16S rRNA gene of strain NK660 was amplified by polymerase chain reaction (PCR) and submitted to GenBank (accession no. JQ014630.1). Basic local alignment search tool (BLAST) searches revealed that strain NK660 shared high homology with *Streptomyces*, and the phylogenetic tree demonstrated that strain NK660 was affiliated into the genus *Streptomyces* (Fig. 2).

During the early stages of growth, strain NK660 formed round and white colonies, and the mycelium was firmly fixed in the medium. Meanwhile, some colonies were white with radial folds on the surface. When the spores grew to maturity, the colony surface was covered with gray spores. The colony characteristics of strain NK660 grown on various media were observed (Supporting Information Table S1). Gray spores were observed on the surface of the colony and no melanoid pigment was observed. The spores had a spiny surface when observed by an electron microscopy (Fig. 1B). The ability of strain NK660 to utilize various carbon sources was evaluated according to the method of Pridham and Gottlieb (1948). The carbon utilization test results are shown in Table 1. Based on its morphological features, spore shape and carbon utilization profiles, strain NK660 was identified as *S. albulus*.

**Table 1:** Utilization of various carbon sources by *S. albulus* NK660

Carbon source	Utilization
L-arabinose	−
D-xylose	−
D-glucose	+
D-fructose	+
L-rhamnose	−
D-galactose	+
Sucrose	−
Raffinose	−
D-mannitol	+
i-inositol	+
i-inositol	−

+, good growth and positive utilization; −, no or faint growth and negative utilization.

### Structural analysis of ε-PL product from strain NK660

**Figure 3:**
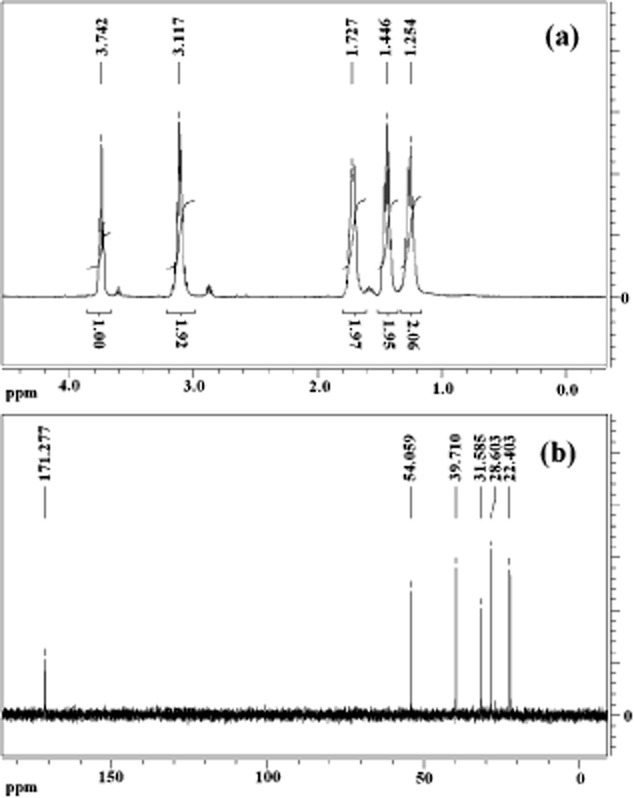
A. ^1^H NMR spectrum of ε-PL produced by *S. albulus* NK660.B. ^13^C NMR spectrum of ε-PL produced by *Streptomyces. albulus* NK660.

**Figure 4:**
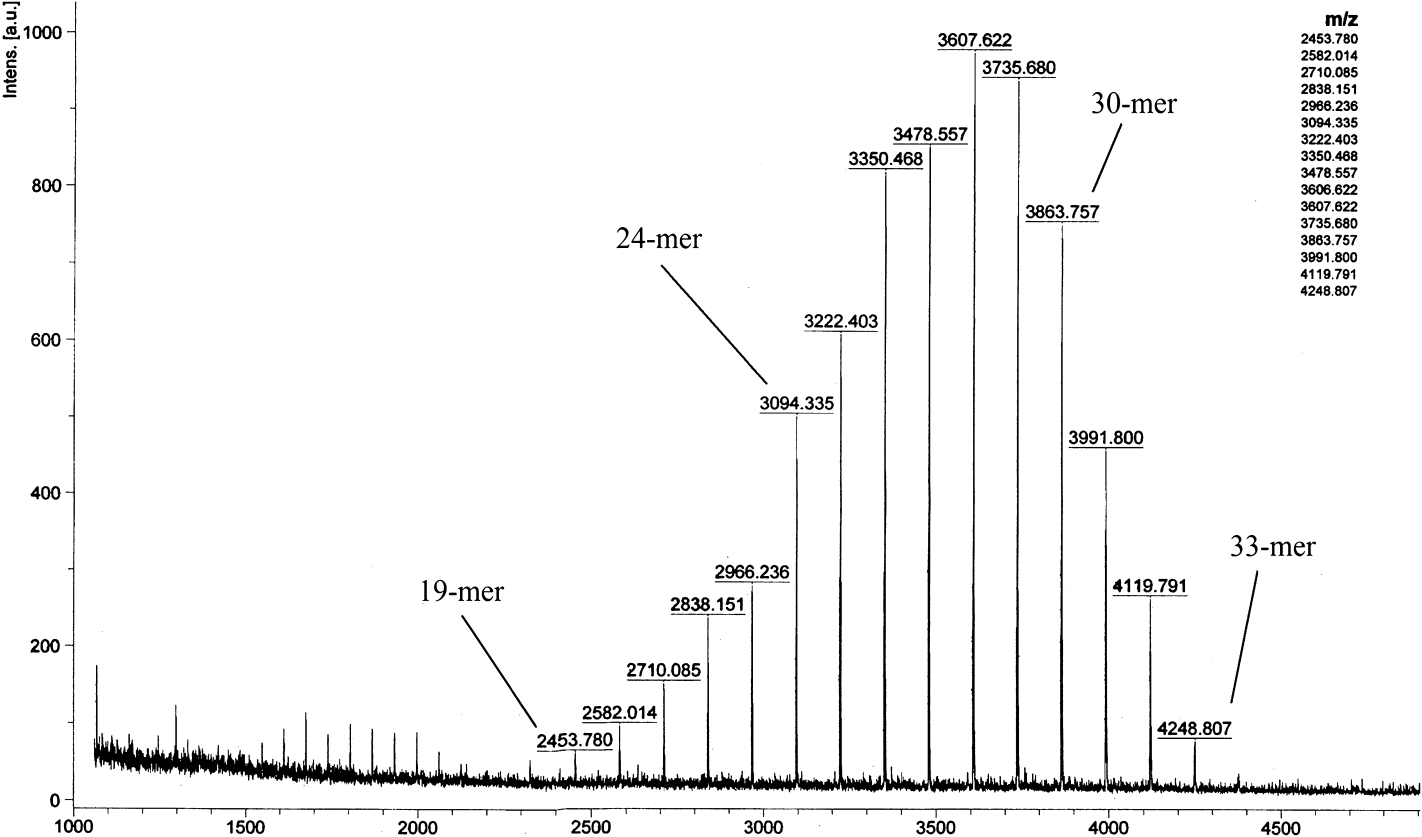
MALDI-TOF MS spectrum of the purified ε-PL product from *S. albulus* NK660.

The ^1^H NMR and the ^13^C NMR spectra of the ε-PL product from strain NK660 are shown in Fig. 3 and consistent with that of the ε-PL standard. The NMR spectra of the ε-PL standard are shown in Supporting Information Fig. S3. The relative molecular mass distribution of ε-PL product from strain NK660 determined by MALDI-TOF MS is shown in Fig. 4. The interval between each peak is 128, which is the molecular weight of a single lysine monomer after dehydration. These data showed that the molecular weight range of the ε-PL produced by strain NK660 (using glycerol as the carbon source) was between 2453–4248 Da, corresponding to the polymerization of 19–33 L-lysine monomers.

The most common ε-PL polymers consist of 24–30 L-lysine monomers. The degree of polymerization and the molecular weight of ε-PL differ among various ε-PL-producing strains. For example, *S. albulus* NBRC14147 synthesized ε-PL with a degree of polymerization of 25–35 L-lysine monomers (Yamanaka *et al*., 2008). Jia and colleagues (2010) reported that a strain of *Streptomyces diastatochromogenes* TUST-2 produced ε-PL with a molecular weight of 1049–4502 Da, corresponding to a degree of polymerization of 8–35 L-lysine monomers, with most of the ε-PL containing 12–20 L-lysine monomers. Nishikawa and Ogawa (2002) reported that *Kitasatospora kifunense* MN-1 and *Epichloe* sp. MN-9 synthesized ε-PL containing 8–17 and 24–29 L-lysine monomers respectively. Takehara and colleagues (2010) reported that *Streptomyces aureofaciens* USE-82 produced short-chain-length ε-PL with a degree of polymerization of 5–20 L-lysine monomers. In the present study, the ε-PL produced by strain NK660 possessed a degree of polymerization consisting of 19–33 L-lysine monomers, with the majority of product containing 24–30 L-lysine monomers.

Shima and colleagues (1984) studied the relationship between the degree of polymerization of ε-PL and antibacterial properties. ε-PL molecules containing less than nine monomers have significantly lower antibacterial potencies (minimum inhibitory concentrations greater than 100 μg ml^−1^). Muto (2009) reported that the antibacterial activity of ε-PL depends on the size of the bacterial cells and the ability of ε-PL to adsorb electrostatically to the bacterial cell membrane. It can be speculated that the ε-PLs of diverse chain length show different absorption capabilities for bacterial cells, thus resulting in the observed differences in antibacterial activities. Therefore, the degree of polymerization and the molecular weight of ε-PL are closely related to its antibacterial activity. Takehara and colleagues (2010) reported that ε-PL polymers with the polymerization degree of 5–20 L-lysine monomers can inhibit the growth of larger microbial cell such as *Saccharomyces cerevisiae*. In addition, the different degrees of polymerization of ε-PL may exert different antimicrobial activities on different groups of bacteria when used in food preservation. The ε-PL produced by strain NK660 was a polymer containing 19–33 L-lysine monomers and the main ε-PL product was a polymer containing 24–30 L-lysine monomers. This degree of polymerization differs from other reported ε-PLs, including other species of *S. albulus*, and this indicates that ε-PL synthesis by strain NK660 could possess potential antibacterial activity. Therefore, ε-PL synthesis by strain NK660 may have particular characteristics as an antimicrobial agent.

### Fed-batch fermentation of strain NK660 for production of ε-PL

**Figure 5:**
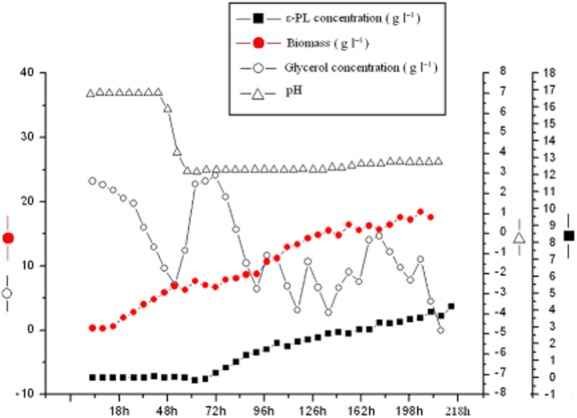
Time curve of process parameters in 30 l fed-batch fermentation of ε-PL-producing *S. albulus* NK660.

In this study, various carbon sources, such as glucose and glycerol, were selected for ε-PL production with strain NK660. As a result, the maximum yield of ε-PL was obtained when using glycerol as the carbon source (Supporting Information Table S2). In previous studies, ε-PL was produced by *S. albulus* NBRC14147 and S*treptomyces roseoverticillatus* MN-10 with glucose as the carbon source (Yamanaka *et al*., 2008; Nishikawa and Kobayashi, 2009). Glycerol is less expensive than glucose, thus, it is suitable for large-scale production of ε-PL. The ability of strain NK660 to utilize glycerol as the carbon source highlights enormous potential of the strain for industrial production of ε-PL at low costs. The pH in the 30 l fermentation was initially maintained at pH 7.0 by adding ammonia for 48 h, and then the pH was allowed to reduce naturally to pH 4.0, although ammonia was still required to maintain this value. In the initial stages of fermentation, the consumption rate of the glycerol was low, but consumption increased rapidly during 18 h to 48 h. Glycerol concentration in the fermentation was kept between 5–15 g l^−1^. Initially, the bacteria grew rather slowly before logarithmic growth occurred between 9 h and 48 h. After 48 h, cell growth reduced and the pH began to fall to pH 4.0. At 54 h, the pH had dropped to pH 4.0 and ε-PL was detected. ε-PL concentration increased gradually to a final yield of 4.2 g l^−1^. Kahar and colleagues (2001) have proposed that the ε-PL synthesis was divided into two control phases, i.e. cell growth phase with the pH at higher than 5.0 and ε-PL production phase with the pH at about 4. The pH is a key factor affecting the yield of ε-PL. In this study, the pH control strategy was also applied for ε-PL production by strain NK660. Time curve of process parameters in the 30 l fermentation was shown in Fig. 5. In the future, optimization of the fermentation conditions will be required for further improvement of ε-PL yields to meet the practical industrial applications.

### Cloning of *pls* gene from strain NK660

**Figure 6:**
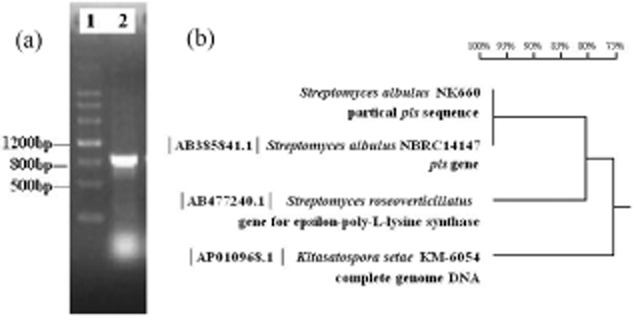
A. Electrophoresis analysis of the *pls* fragment obtained by PCR using the primers designed based on the previously reported *pls* gene sequence. Lane 1, DNA Marker III; lane 2, the 800 bp fragment amplified from strain NK660.B. The homology comparison of the 780 bp nucleotide sequence of strain NK660 to the *pls* gene found in *S. albulus* NBRC14147, *S. roseoverticillatus* MN-10 and *Kitasatospora setae* KM-6054.

According to the *pls* sequence from *S. albulus* NBRC14147 (accession no. AB385841) and other *Streptomycete* strains (Yamanaka *et al*., 2008; Nishikawa and Kobayashi, 2009), a pair of primers was designed to amplify the partial *pls* gene (Supporting Information Fig. S1). A 780 bp fragment was obtained from strain NK660 (Fig. 6A), and the results from NCBI BLAST revealed that the fragment showed 100% and 84% similarity, respectively, to the *pls* gene of *S. albulus* NBRC14147 and *S. roseoverticillatus* MN-10 (Fig. 6B). Based on the 780 bp nucleotide sequence and the *pls* gene of *S. albulus* NBRC14147, multiple primers were designed to conduct genomic DNA walking (Supporting Information Table S3). Finally, a 5283 bp nucleotide sequence containing the intact *pls* gene of strain NK660 was identified. The *pls* gene sequence of strain NK660 has been deposited in the GenBank database under accession no. JN896703.

**Figure 7:**
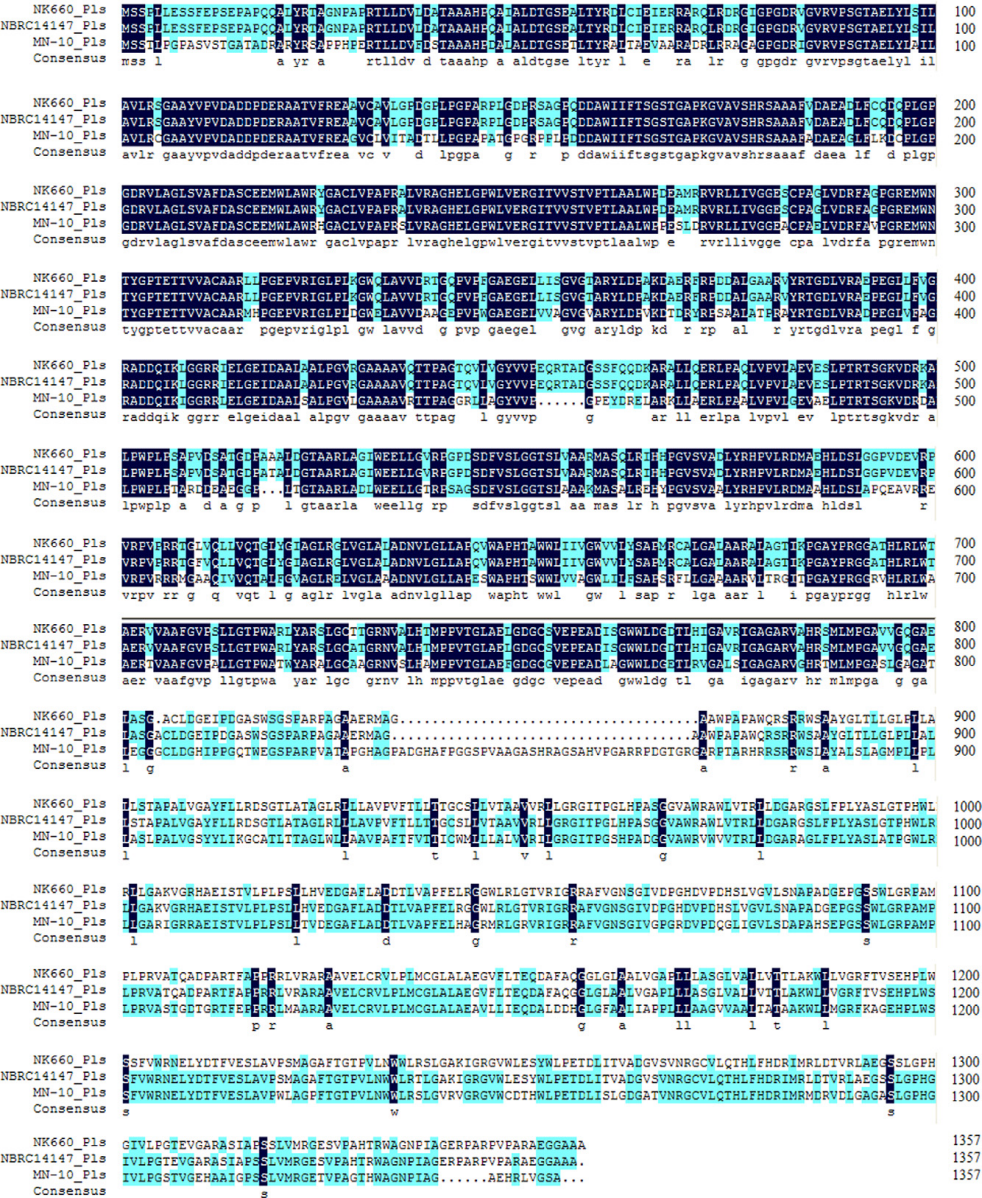
The alignment of the amino acid sequence of ε-PL synthetase from *S. albulus* NK660 with two previously reported ε-PL synthetases from *S. albulus* NBRC14147 and *S. roseoverticillatus* MN-10.

As shown in Fig. 7, the amino acid sequence of the ε-PL synthetase encoded by the *pls* gene of strain NK660 was aligned with that of ε-PL synthetases from other *Streptomycete* strains using the Clustal X software (Conway Institute UCD, Dublin, Ireland).

### Heterologous expression of *pls* gene in *S. lividans*

**Figure 8:**
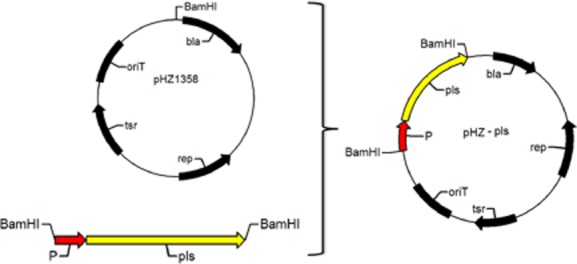
A schematic diagram of the construction of the expression vector pHZ-pls.

In previous studies, we tried to express the *pls* gene of strain NK660 in *E. coli*, but we failed. In this study, *S. lividans* ZX7 was chosen as a host strain for heterologous expression of *pls* gene. Plasmid pHZ1358 contains a pIJ101 replicon, thus, it can stably exist in *S. lividans* ZX7 when thiostrepton is added (Sun *et al*., 2009). Based on the pHZ1358, the recombinant pHZ-pls vector harbouring *pls* gene from strain NK660 was constructed (Fig. 8) and transformed into *E. coli* ET12567. The pHZ-pls was transformed into *S. lividans* ZX7 by conjugation, resulting in the construction of the recombinant strain *S. lividans* ZX7-pls. Seven colonies were picked up and confirmed by colony PCR (Supporting Information Fig. S4). One of these positive recombinants, named *S. lividans* ZX7-pls, was chosen for further studies.

To test whether production of ε-PL during fermentation inhibits cell growth, the growth kinetics of *S. lividans* ZX7 cells harbouring pHZ-pls or the empty vector pHZ1358 were compared. No growth inhibition was observed for cells expressing Pls (pHZ-pls). These cells carrying pHZ-pls showed the same growth profile as those cells carrying pHZ1358. Fermentation of *S. lividans* ZX7-pls was performed in M3G medium with 2.5 μg ml^−1^ thiostrepton at 30°C for 120 h. The culture supernatant was positively reacted with Dragendorff reagent, leading to the formation of sediment (Supporting Information Fig. S2). The ε-PL polymer produced by *S. lividans* ZX7-pls consisted of different numbers of L-lysine monomers. These results indicated that the *pls* gene was functionally expressed in *S. lividans* ZX7, and that the ε-PL was successfully synthesized in *S. lividans* ZX7. The total cell protein from *S. lividans* ZX7-pls was incubated with L-lysine and ATP, and the ε-PL product was extracted from the reaction mixture and quantified as an indicator of the Pls activity, indicating that the Pls activity in *S. lividans* ZX7-pls was much lower than that in *S. albulus* NK660. In this study, although the amount of the ε-PL produced by *S. lividans* ZX7-pls was lower than *S. albulus* NK660, the heterologous expression of the ε-PL synthetase gene in *S. lividans* ZX7 would open new perspectives, not only for exploring the biosynthetic mechanisms of ε-PL in cells, but also for investigating the key factors that affect the yield and the molecular weight of ε-PL. This is the first report of the functional expression of the ε-PL synthetase gene in *S. lividans*. Optimization of the fermentation conditions and the gene expression systems will further improve the ε-PL production efficiency in *S. lividans* ZX7-pls. For example, the use of a strong promoter instead of the native promoter will accelerate the transcription rate of the *pls* gene in *S. lividans* ZX7, resulting in the production of more Pls molecules. This work is currently in progress.

In general, the chain length of a polymer might be attributed to the biosynthetic machinery or the degrading enzymes. In this study, the polymerization degree of ε-PL produced by strain NK660 was lower than that of ε-PL produced by *S. albulus* NBRC14147, even if their Pls had more than 99% similarity. Yamanaka and colleagues (2010) reported that the chain length of ε-PL is not determined by ε-PL-degrading enzymes. In another study, in vitro-produced ε-PL had a chain length ranging from 3 to 17 L-lysine monomers, while in vivo-produced ε-PL consisted of 25–35 L-lysine monomers (Yamanaka *et al*., 2008), which suggested that the polymerization degree of ε-PL would also be related to the intracellular environment of the ε-PL-producing strain. Expression of Pls in other strains is a feasible approach to investigate the effect of intracellular environment on the chain length of ε-PL. In this study, heterologous expression of Pls was achieved with *S. lividans* ZX7, suggesting that heterologous expression of *pls* is an important way for elucidating the biosynthetic mechanisms of ε-PL.

*S. albulus* NK660 and *S. albulus* NBRC14147 produce ε-PL polymers with different chain lengths and with different molecular size distributions. Yamanaka and colleagues (2008) have clarified the catalytic mechanisms of the ε-PL synthetase in *S. albulus* NBRC14147. Therefore, in-depth study of the structure of the ε-PL synthetase in strain NK660 and the ε-PL biosynthetic pathway is significant as this will clarify the polymerization mechanisms involved in the chain-length diversity of ε-PL.

## Materials and methods

### Screening for strains producing ε-PL

Soil samples which were collected from different places of China (Hebei, Liaoning, Henan and Fujian provinces) were mixed with a few CaCO_3_ and air-dried in the shade for 10 days. A 10-fold dilution series of soil suspensions were prepared and 100 μl of each soil suspension supernatant was spread onto a synthetic glycerol (SG) medium (Nishikawa and Ogawa, 2002) supplemented with 150 mg l^−1^ of K_2_Cr_2_O_7_. The compositions of SG medium were as follows (per liter): 10 g of glycerol, 0.1 g of yeast extract, 0.68 g of NaH_2_PO_4_, 0.25 g of KH_2_PO_4_, 0.25 g of MgSO_4_·7H_2_O, 0.66 g of (NH_4_)_2_SO_4_, 0.05 g of ZnSO_4_·7H_2_O and 0.01 g of FeSO_4_·7H_2_O, pH of 7.0. After incubation at 28°C for 7 days, actinomycete colonies were selected from the SG agar plate and inoculated onto an SG agar indicator plate containing 0.002% of methylene blue. After incubation at 28°C for 3 days, the colonies forming concentric zones were chosen based on the principle described by Nishikawa and Ogawa (2002). Colonies producing zones of clearance were inoculated into liquid SG medium and incubated for 1 week. Then, the culture supernatant was detected by Dragendorff reagent (Shima and Sakai, 1977), and the positive results appeared. Dragendorff reagent consisted of 5 ml of solution 1 (0.8 g of pentahydrate bismuth nitrate, 40 ml of distilled water, 10 ml of glacial acetic acid) and 5 ml of solution 2 (8.0 g of potassium iodide, 20 ml of distilled water) made up to 100 ml with distilled water.

### Identification of ε-PL-producing strain

Total genomic DNA was extracted from strain NK660 by a standard phenolic extraction procedure (Sambrook and Russel, 2001). The 16S rRNA gene of strain NK660 was amplified by PCR from the genomic DNA using the universal primers, 27f (5′-AGAGTTTGATCMTGGCTCAG-3′) and 1492r (5′-CGGYTACCTTGTTACGACTT-3′) (Weisburg *et al*., 1991). The PCR reaction was performed in a GeneAmp PCR system 9700 thermocycler (Applied Biosystems, Foster City, CA, USA) with the following cycling profile: initial denaturation at 94°C for 5 min, 30 cycles of denaturation at 94°C for 1 min, annealing at 55°C for 1 min, and extension at 72°C for 1.5 min, final extension at 72°C for 8 min. The PCR product was cloned into a pMD18-T vector (TaKaRa, Dalian, Liaoning, China) and sequenced. The determined sequence was compared with those available in the GenBank database using the NCBI BLAST program. Multiple alignments of sequences, construction of a neighbour-joining phylogenetic tree with the Kimura 2-parameter model and a bootstrap analysis for evaluation of the phylogenetic topology were accomplished using Clustal X program (Thompson *et al*., 1997) and the MEGA 4.0 package (Tamura *et al*., 2010).

The culture characteristics of strain NK660 were investigated in different media and the surface features of spores were observed by electron microscopy; the results were compared with that described in the 8^th^ edition of Bergey's manual. Then, a variety of carbon utilization experiments were conducted according to the method of Pridham and Gottlieb (1948). The compositions of carbon utilization medium were as follows (per liter): 2.64 g of (NH_4_)_2_SO_4_, 2.38 g of KH_2_PO_4_, 5.65 g of K_2_HPO_4_·3H_2_O, 1.00 g of MgSO_4_·7H_2_O, 0.0064 g of CuSO_4_·5H_2_O, 0.0015 g of FeSO_4_·7H_2_O, 0.0079 g of MnCl_2_·4H_2_O, 0.0011 g of ZnSO_4_·7H_2_O and 10 g of a particular carbon source. The tested carbon sources included L-arabinose, D-xylose, D-glucose, D-fructose, L-rhamnose, D-galactose, sucrose, raffinose, D-mannitol, i-inositol and salicin.

### Structural analysis of ε-PL product from strain NK660

The structure of the purified ε-PL was determined by ^1^H NMR, ^13^C NMR and MALDI-TOF MS. NMR spectra were recorded on a Bruker AV-400 spectrometer (Bruker Corporation, Billerica, MA, USA) at 400 MHz. The polymers were prepared at 10 mg ml^−1^ in D_2_O, and chemical shifts were measured at 25°C in 5 mm diameter tubes (Maeda *et al*., 2005; Hirohara *et al*., 2006). ε-PL samples were analyzed by MALDI-TOF MS with an Autoflex III TOF/TOF 200 (Bruker Corporation) instrument. 2, 5-Dihydroxybenzoic acid (DHB) was used as the matrix (Nishikawa and Kobayashi, 2009).

### Fed-batch fermentation of strain NK660 for production of ε-PL

A synthetic medium modified from M3G medium (Kahar *et al*., 2001), which contained 10 g of (NH_4_)_2_SO_4_, 30 g of glycerol, 7 g of peptone, 1.36 g of KH_2_PO_4_, 0.8 g of K_2_HPO_4_·3H_2_O, 4 g of MgSO_4_·7H_2_O, 0.04 g of ZnSO_4_·7H_2_O, 0.03 g of FeSO_4_·7H_2_O per liter, pH of 7.0, was used for the 30 l fed-batch fermentation. The medium was sterilized at 115°C for 30 min. A loopful of NK660 spores were inoculated into Luria-Bertani medium (Sambrook and Russel, 2001) and cultured at 30°C, 200 r.p.m. for 24 h. Then, 1.8 l of this seed culture was inoculated into a 30 l fermenter containing 18 l of modified M3G medium. Glycerol concentration during fermentation was monitored using an enzymatic kit (Nanjing Jiancheng Bioengineering Institute, Nanjing, Jiangsu, China). The feeding solution contained 180 g l^−1^ of glycerol and 9 g l^−1^ of ammonium sulphate, and feeding was commenced via a peristaltic pump when the glycerol concentration fell below 5 g l^−1^. Feeding was terminated when the glycerol concentration reached 15 g l^−1^. The stirring rate was 180 r.p.m. Real-time pH changes were monitored using a pH electrode, and ammonia was added to maintain the pH at pre-determined levels. Meanwhile, dissolved oxygen (DO) was detected using a DO electrode, and aeration varied from 0.5 to 2.5 vvm.

After fermentation, the culture was centrifuged at 8000 r.p.m. for 20 min to pellet the mycelia. The supernatant was collected and adjusted to a pH of 8.0 with 6 M of NaOH, and then filtered to eliminate the precipitate. Then, the supernatant was mixed with weak acid cation resin D152 to collect ε-PL, as the resin has high affinity for ε-PL. The resin was washed with distilled water to remove any unabsorbed impurities, while ε-PL was eluted with 0.4 M of HCl. Subsequently, the eluate was adjusted to neutral with 6 M of NaOH, filtered by activated carbon to remove pigment and then dialysed overnight for desalination. The eluate was freeze-dried and ε-PL yield was assessed according to the method described in Itzhaki (1972).

### Cloning of the *pls* gene from strain NK660

Based on the conserved sequence of *pls* gene of ε-PL-producing *Streptomyces* strains, a pair of primers was designed to amplify a fragment of the *pls* gene of *S. albulus* NK660 (Supporting Information Fig. S1). PCR amplification was performed using TaKaRa LA Taq and GC buffer II (TaKaRa). The annealing temperature was determined according to the primer, and elongation time was determined according to the length of expected fragment. Primers to clone the *pls* fragment were designed by Primer 5.0 software (PREMIER Biosoft International, Palo Alto, CA, USA). The primers designed for cloning of the intact *pls* gene by genome walking are listed in Supporting Information Table S3.

### Heterologous expression of *pls* gene in *S. lividans*

For the expression of *pls* gene in *S. lividans*, the intact *pls* gene with its native promoter was amplified with forward primer 5′-CGCGGATCCTGCTCTTTGGTCTGGTGGGAATC-3′ and reverse primer CGCGGATCCACGGTGTCGTGGGCGTAGGT. The PCR products were digested with *Bam*HI and subcloned into a *Streptomyces* expression vector pHZ1358 (Sun *et al*., 2009) to generate pHZ-pls. The pHZ-pls was transformed into *E. coli* ET12567 and then transferred into *S. lividans* ZX7 by biparental conjugation (Lin *et al*., 1993). The conjugation mixture was spread on mannitol soy flour agar plates. The plates were incubated for 16–21 h at 30°C and then overlaid with 1.5 ml of sterile water containing 5 μg ml^−1^ of thiostrepton and 30 μg ml^−1^ of nalidixic acid. After incubation for 5 days at 30°C, the transformants were picked up and detected by colony PCR. One positive transformant (named *S. lividans* ZX7-pls) was used for the synthesis of ε-PL. Subsequently, the fermentation experiments were conducted with *S. lividans* ZX7-pls at 30°C for 120 h in M3G medium containing 2.5 μg ml^−1^ of thiostrepton. The ε-PL product was detected by Dragendorff reagent as described earlier.

## Supporting information

**Fig. S1.** A pair of primers designed for amplifying the partial *pls* gene of *S. albulus* NK660. **Fig. S2.** Detection of ε-PL production in *S. lividans* by Dragendorff reagent. **Fig. S3.** A. 1H NMR spectrum of a reference standard of ε-PL. B. 13C NMR spectrum of a reference standard of ε-PL. **Fig. S4.** A. The construction of pHZ-pls confirmed by restriction enzyme digestion. B. The PCR detection of the pHZ-pls in *S. lividans* ZX7. **Table S1.** Culture characteristics of *S. albulus* NK660. **Table S2.** Effects of different carbon sources on cell dry weight, final pH and ε-PL yield. **Table S3.** The primers used for cloning of the *pls* gene from *S. albulus* NK660 by genome walking.
