# Supplementary material for: Cloning of ε-poly-L-lysine (ε-PL) synthetase gene from a newly isolated ε-PL-producing Streptomyces albulus NK660 and its heterologous expression in Streptomyces lividans
Source: Microb Biotechnol. 2014 Jan 14;7(2):155–64. doi: 10.1111/1751-7915.12108 (PMC3937719; doi:10.1111/1751-7915.12108)
Supplement: Supplementary file 1 — Fig. S1. A pair of primers designed for amplifying the partial pls gene of S. albulus NK660. Fig. S2. Detection of ε-PL production in S. lividans by Dragendorff reagent. Fig. S3. A. 1H NMR spectrum of a reference standard of ε-PL. B. 13C NMR spectrum of a reference standard of ε-PL. Fig. S4. A. The construction of pHZ-pls confirmed by restriction enzyme digestion. B. The PCR detection of the pHZ-pls in S. lividans ZX7. Table S1. Culture characteristics of S. albulus NK660. Table S2. Effects of different carbon sources on cell dry weight, final pH and ε-PL yield. Table S3. The primers used for cloning of the pls gene from S. albulus NK660 by genome walking. [file mbt20007-0155-sd1.doc]

**Table S1.** Culture characteristics of *Streptomyces albulu* NK660

| Agar medium | Aerial mycelium | Substrate mycelium | Soluble pigment |
| --- | --- | --- | --- |
| Tyrosine agar | Poor, white, tight and small colony | Light yellow | No |
| Inorganic salts-starch | White and gray in the center of the colony, large and rough surface | Light yellow to gray | No |
| Glucose-asparagine | White colony, and with gray edge | Light yellow | No |
| Czapeck’s | White or grayish, tight and small colony | White to light yellow | No |
| Gause No.1 | White in the edge and center, gray in the rest part | White to light yellow | No |

**Table S2.** Effects of different carbon sources on cell [dry weight](http://dict.cnki.net/dict_result.aspx?searchword=菌体干重&tjType=sentence&style=&t=dry+cell+weight), final pH, and ε-PL yield

|  | glucose | glycerol | soluble starch | sucrose | maltose | lactose | dried corn steep liquor powder |
| --- | --- | --- | --- | --- | --- | --- | --- |
| final pH | 3.15 | 3.11 | 3.99 | 7.47 | 3.68 | 7.74 | 4.13 |
| cell dry weight (g/L) | 3.91 | 3.61 | 8.9 | 3.34 | 2.61 | 1.98 | 1.28 |
| ε-PL yield (g/L) | 0.56 | 0.91 | 0.43 | 0.039 | 0.052 | 0.040 | 0.050 |

**Table S3.** The primers used for cloning of the *pls* gene from *Streptomyces albulu* NK660 by genome walking

| P485-F | ACGACGCCTGGATCATCTTCACCTC |
| --- | --- |
| P646-F | ACGCCTCCTGCGAGGAGATGTGGCT |
| P769-F | ATCACCGTCGTCTCCACCGTGCCCAC |
| P1F | ATGTCGTCGCCCCTTCTCGAATCGT |
| P1320F | CACCCAGGTGCTGGTCGGCTACGTCGTTC |
| P1320R | GAACGACGTAGCCGACCAGCACCTGGGTG |
| P2688R | CAGCCAGTGGTCAGGAGCGTGAAGA |
| P485-F | ACGACGCCTGGATCATCTTCACCTC |
| P646-F | ACGCCTCCTGCGAGGAGATGTGGCT |
| P769-F | ATCACCGTCGTCTCCACCGTGCCCAC |
| P1492-F | TCCGGCAAGGTCGACCGC |
| P1492-R | GCGGTCGACCTTGCCGGA |
| P2130-F | CGTCGCCGCCTTCGGCGTCCCC |
| P2130-R | GGGGACGCCGAAGGCGGCGACG |
| P2302-F | GGCTGGTGGCTCGACGGCGA |
| P2302-R | TCGCCGTCGAGCCACCAGCC |
| P3851-a-R | CGCAWGATCCGGTCGTGGAA |
| P3833-a-R | TGGAASAGGTGSGTCTGCAGGAC |
| pls-200-F | GGGAGTGGTGCCGAAAAC |
| pls-4100R | GTGTCGTGGGCGTAGGTG |
| 2100F | GTCACCGGCCTTGCCGAACT |
| 3400R | GGAGCGCAGCCACCAGTTCA |
| PLS-F-ORF13 | CGATGAGGTCGGCGTGAGA |
| P300R | ACGGCGAGGATGGACAGGTA |
| pls-R-ORF14 | AAGCCCTCGTTGAGCCAGATGT |
| pls-3200-F | CCCCTGTGGTCGTCGTTCGT |
| pls-3200-R | ACGAACGACGACCACAGGGG |
| 1965F | CATCGTCGGCTGGGTGGTG |
| 2640R | GCTGTCGCGGAGCAGGAAGT |
| 2707R | CGGTGGTCAGGAGCGTGAAG |


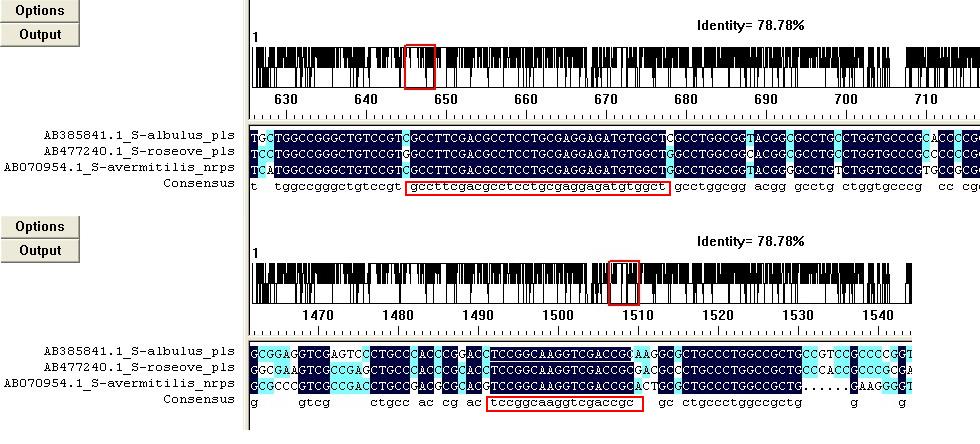


**Fig. S1.** A pair of primer designed for amplifying the partial *pls* gene of *Streptomyces albulu* NK660. Based on the conserved sequence of the *pls* gene of three strains, a pair of primer was designed to amplify the fragment of the *pls* gene of *S. albulu* NK660.


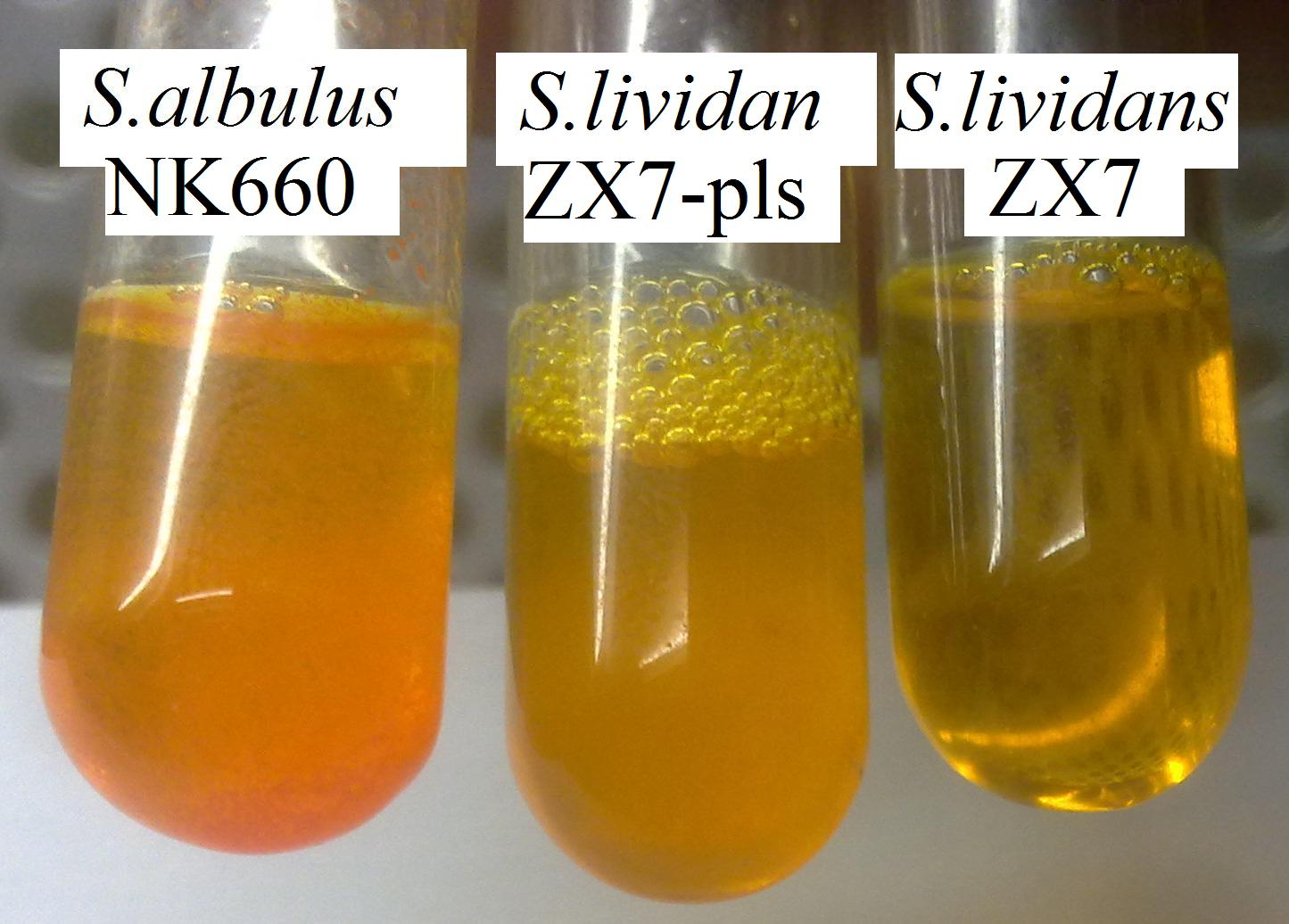


**Fig. S2.** Detection of ε-PL production in *S. lividans* by Dragondoff reagent. The culture supernatant of *S. lividans* ZX7-pls was detected by Dragondoff reagent and positive results [appeared](http://dict.cnki.net/dict_result.aspx?searchword=出现&tjType=sentence&style=&t=appeared).


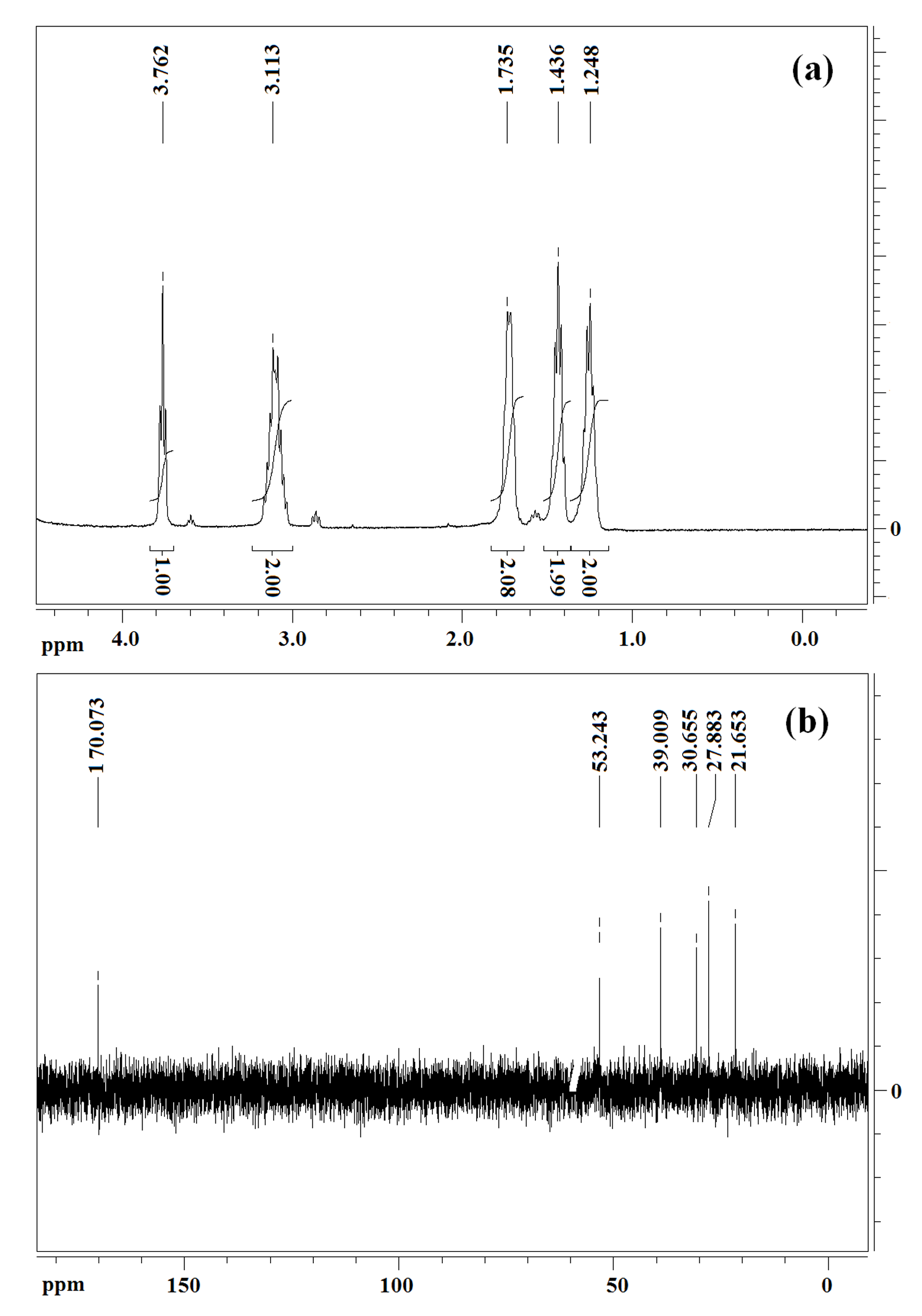


**Fig. S3.** ^1^H NMR spectrum (a) and ^13^C NMR spectrum (b) of a reference standard of ε-PL.


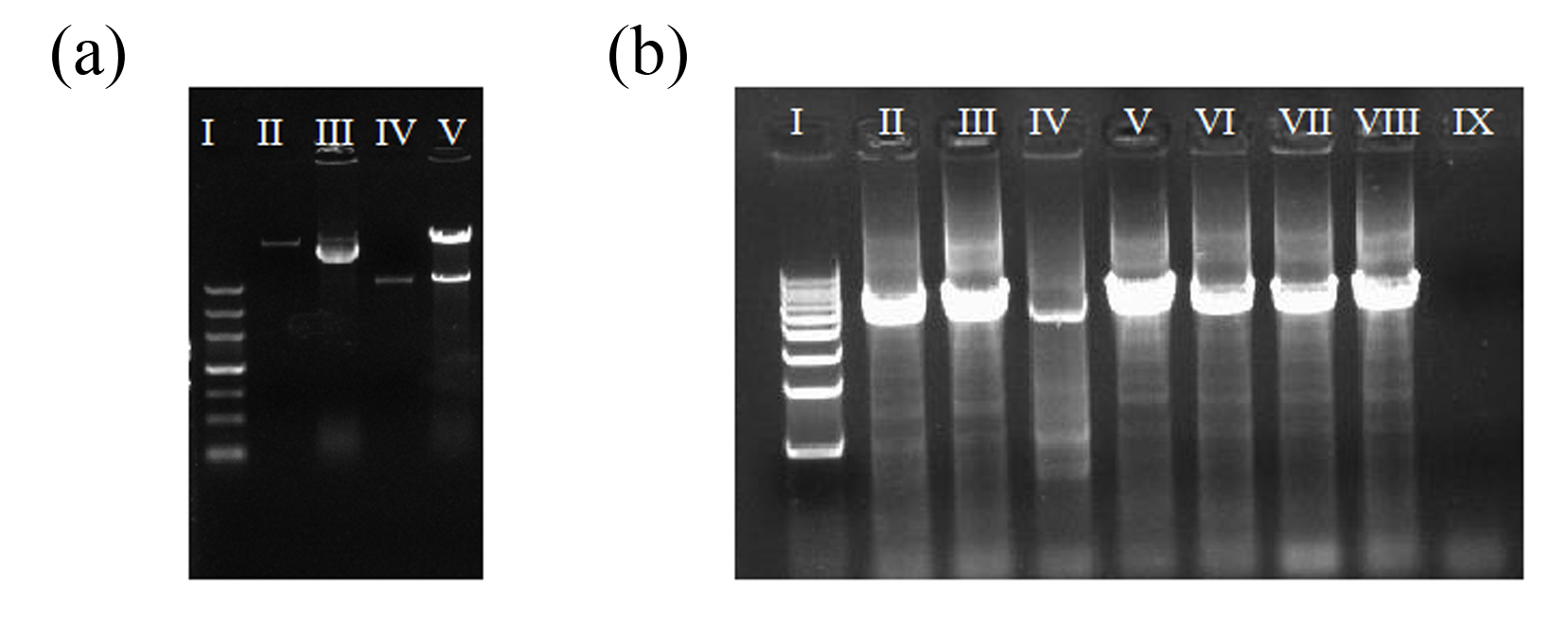


**Fig. S4.** (a) The construction of pHZ-pls confirmed by restriction enzyme digestion. Lane I, Marker III; lane II, pHZ1358 digested by *Bam*HI; lane III, pHZ-pls; lane IV, *pls* fragment; lane V, pHZ-pls digested by *Bam*HI. (b) The PCR detection of the pHZ-pls in *S. lividans* ZX7. The full-length *pls* gene was amplified from the transformants. Lane I, DNA ladder; lane II-VIII, *S. lividans* ZX7 positive transformants harboring pHZ-pls; lane IX, *S. lividans* ZX7.
